# Cell type–specific dissection of cell death programs during ovarian aging

**DOI:** 10.1093/bib/bbag399

**Published:** 2026-07-30

**Authors:** Ruizhe Wang, Di Wu, Sheng Li, Ying Yang, Hui Xue

**Affiliations:** Department of Gynecology, The First Affiliated Hospital of China Medical University, No. 155 Nanjing North Street, Heping District, Shenyang 110001, Liaoning Province, P.R. China; Department of Cardiology, The First Affiliated Hospital of China Medical University, No. 155 Nanjing North Street, Heping District, Shenyang 110001, Liaoning Province, P.R. China; Zhongnan Hospital of Wuhan University, No. 169 Donghu Road, Wuchang District, Wuhan 430060, Hubei Province, P.R. China; Department of Operating Room, The First Affiliated Hospital of China Medical University, No. 155 Nanjing North Street, Heping District, Shenyang 110001, Liaoning Province, P.R. China; Department of Gynecology, The First Affiliated Hospital of China Medical University, No. 155 Nanjing North Street, Heping District, Shenyang 110001, Liaoning Province, P.R. China

**Keywords:** ovarian aging, programmed cell death, transcriptome deconvolution, multi-task deep learning

## Abstract

The accurate deconvolution of bulk transcriptomes typically confounds stable physical cell identities with dynamic physiological states, limiting our understanding of complex microenvironmental processes such as ovarian aging. To overcome this, we present DeepMCD, an end-to-end multi-task deep learning framework designed to simultaneously deconvolve cell-type proportions and programmed cell death (PCD) compositional fractions from standard bulk RNA-seq data. By mapping high-dimensional expression profiles into a shared, tokenized latent space, DeepMCD employs a Transformer-based cross-task attention mechanism to explicitly leverage cellular morphological context for calibrating PCD predictions. Concurrently, an adaptive uncertainty-weighting loss ensures balanced optimization, effectively mitigating negative transfer. Extensive benchmarking demonstrates that DeepMCD significantly outperforms state-of-the-art single-task algorithms. Through rigorous ablation and interpretability analyses, we computationally substantiate the biological premise that functional death states are heavily reliant on specific cell-type contexts. Applying DeepMCD to real-world mouse ovarian aging cohorts, we reconstructed a cell-type-specific PCD landscape, bypassing the need for costly single-cell sequencing. Specifically, we identified an age-associated increase in inflammatory and lytic death modalities, together with a strong association between macrophage enrichment and pyroptosis during ovarian aging. Crucially, the DeepMCD-derived PCD fractions exhibit profound divergent correlations with core ovarian fibrosis-related genes. These computationally extracted signatures may serve as cost-effective candidate digital biomarkers for evaluating ovarian fibrosis and reproductive senescence. Ultimately, DeepMCD provides a highly interpretable, robust, and scalable computational tool for bulk RNA-seq data decoding.

## Introduction

Ovarian aging is a primary driver of female reproductive decline, characterized by a decrease in the number of primordial follicles and a deterioration of oocyte quality [1, 2]. Beyond infertility, the premature or physiological decline of ovarian function is associated with significant social and medical burdens, including an increased risk of cardiovascular disease, osteoporosis, and cognitive impairment [3]. During this process, PCD acts as a highly regulated biological mechanism essential for maintaining tissue homeostasis and eliminating damaged cells [4]. While apoptosis was long considered the primary mechanism of follicular atresia [5], recent research has uncovered a diverse array of non-apoptotic PCD forms, such as pyroptosis, ferroptosis, and autophagy-dependent cell death [6, 7]. These multifaceted pathways play fundamental roles in the development of various age-related pathologies by regulating inflammation, oxidative stress, and metabolic homeostasis [8]. Previous studies have demonstrated that localized cell death is a critical driver of follicular atresia and stromal fibrosis [9]. However, a comprehensive characterization of how an expanded spectrum of diverse PCD modalities maps to specific ovarian lineages has remained largely unexplored, leaving a holistic understanding of how these diverse PCD mechanisms synergistically contribute to ovarian aging incomplete.

Currently, defining this cell-type-specific PCD landscape heavily relies on single-cell RNA sequencing (scRNA-seq) [10]. While scRNA-seq offers unprecedented cellular resolution, its widespread application in large-scale clinical and preclinical cohorts is fundamentally constrained by high costs, complex tissue dissociation protocols, and the potential loss of rare fragile cells (such as highly apoptotic or necroptotic populations) [11, 12]. In contrast, bulk RNA sequencing remains the most abundant and cost-effective transcriptomic modality, preserving the global macro-environmental profile [13]. Yet, bulk RNA-seq only provides an averaged ‘smoothie’ of gene expression, completely confounding stable physical cell fractions with dynamic physiological activities.

Computational deconvolution algorithms have emerged as a powerful alternative to computationally resolve bulk transcriptomes. Traditional methods (e.g., CIBERSORTx, EPIC) [14, 15] and recent deep learning-based approaches (e.g., Scaden, DeSide) [16–18] have achieved remarkable success in estimating physical cell-type proportions. In parallel, scoring algorithms like GSVA or ssGSEA are widely used to estimate the overall enrichment of PCD-related pathways at the tissue level [19, 20]. In addition, pathway-activity inference frameworks such as decoupleR and PROGENy provide complementary approaches for assessing pathway activity, although they are not designed to estimate cell-type-specific compositional fractions in an end-to-end deconvolution setting. However, these existing computational frameworks treat cell-type composition and physiological state activity as mutually exclusive, isolated tasks. Biologically, physical cell identities and functional states are deeply intertwined—for instance, inflammatory pyroptosis is predominantly executed by macrophages rather than stromal cells [21]. Current single-task deconvolution models fail to leverage this crucial biological prior, rendering them incapable of determining the precise mapping between specific cell types and their corresponding death programs.

To bridge this methodological gap, we propose DeepMCD, a novel multi-task deep learning architecture designed for the simultaneous and interactive deconvolution of cell-type proportions and PCD state activities from standard bulk RNA-seq data. Instead of employing isolated prediction networks, our model maps high-dimensional bulk expression profiles into a shared, tokenized latent space. By introducing highly specialized class tokens ([CLS]) and a Transformer-based cross-task attention mechanism [22], alongside an adaptive uncertainty-weighting loss [23], our framework explicitly models the synergistic dependencies between physical cellular components and dynamic functional states. This design allows the network to dynamically utilize cell-type context as a biological guide to accurately calibrate PCD pattern predictions, heavily mitigating the negative transfer often seen in complex multi-task learning.

In this study, we comprehensively benchmarked our algorithm on large-scale scRNA-seq-derived pseudo-bulk datasets, demonstrating superior accuracy over existing single-task baselines. Through rigorous attention-masking ablation studies, we computationally validated the fundamental hypothesis that cell-type contextual information significantly enhances the predictive fidelity of cell death programs. Finally, we applied our model to real-world bulk transcriptomic profiles of mouse ovarian aging. Bypassing the need for costly single-cell experiments, our algorithm successfully reconstructed the high-resolution, cell-type-specific PCD network—identifying macrophage-driven pyroptosis as a primary orchestrator of microenvironmental deterioration. Furthermore, we demonstrated that DeepMCD-derived PCD fractions strongly correlate with ovarian fibrosis-related genes (e.g., Tgfb1, Col1a1, Col1a2, Fn1), establishing them as highly accurate, cost-effective candidate digital biomarkers for evaluating ovarian fibrosis and reproductive senescence.

## Materials and methods

### Overview of DeepMCD

**Figure 1:**
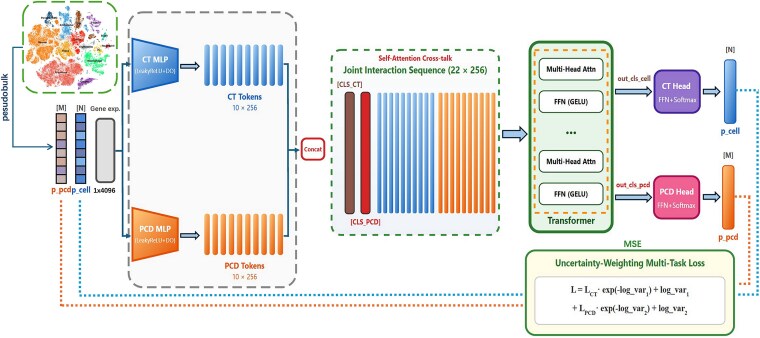
Schematic overview of the DeepMCD architecture. Normalized bulk expression profiles are decoupled into CT and PCD tokens via dual-branch MLPs. A transformer encoder processes the joint interaction sequence to capture cross-task dependencies, and the entire framework is optimized end-to-end via an uncertainty-weighting multi-task loss.

To bridge the methodological gap of simultaneously deconvolving physical cellular identities and dynamic physiological states from bulk transcriptomics, we propose DeepMCD (Deep Multi-task Cell death Deconvolution). DeepMCD is an end-to-end multi-task deep learning framework characterized by dual-branch latent tokenization and a Transformer-based cross-task attention mechanism (Fig. 1).

The overall workflow of DeepMCD consists of four major computational modules:

First, to overcome the lack of dual-labeled bulk data, high-resolution single-cell RNA sequencing (scRNA-seq) data is utilized to construct a vast array of pseudo-bulk mixtures, generating reliable ground-truth proportions for both cell types (CT) and PCD programs. Second, normalized bulk gene expression vectors ($1\times 4096$) are fed into two parallel Multi-Layer Perceptron (MLP) encoders. Instead of using a shared backbone, these dual MLPs decouple the entangled high-dimensional input into discrete latent representations, specifically 10 CT tokens and 10 PCD tokens (each shaped $1\times 256$). Third, to explicitly model the synergistic dependencies between cell types and their death patterns, two learnable class tokens ([CLS_CT] and [CLS_PCD]) are initialized and prepended to the feature tokens, forming a joint interaction sequence ($22\times 256$). This sequence is processed by a Transformer encoder, where multi-head self-attention mechanisms enable the PCD representation to dynamically extract contextual cellular identity information. Finally, the enriched [CLS] tokens are decoded by task-specific feed-forward networks (FFN) to predict the respective proportions. The entire framework is optimized using an uncertainty-weighting multi-task loss function to dynamically balance the Mean Squared Error (MSE) of both objectives without manual hyperparameter tuning.

### Data acquisition and Preprocessing

To train and validate the DeepMCD framework, and to subsequently evaluate its clinical applicability, we collected both scRNA-seq and bulk transcriptomic datasets from the Gene Expression Omnibus (GEO) database [24]. The essential information of all scRNA-seq and bulk RNA-seq cohorts included in this study is detailed in Supplementary Table S1.

For the construction of the scRNA-seq reference atlas, datasets including GSE200612, GSE232309, GSE236712, and GSE317144 were aggregated. The scRNA-seq data were processed using the Scanpy (v1.9) Python package. Quality control was enforced by excluding cells with fewer than 300 or more than 7000 detected genes, or with mitochondrial content exceeding 20%. Raw counts were library-size normalized to 10,000 counts per cell and log1p-transformed. Batch effects across different scRNA-seq datasets were corrected using the Harmony algorithm [25], retaining 147,483 high-quality cells. The robust identification of distinct cell types was validated via UMAP projections and cell-type-specific marker gene expression (Supplementary Fig. S1A, B).

Key regulatory genes for 13 named PCD pathways were obtained from curated sources including the KEGG [26], GeneCards [27], Reactome [28], and Molecular Signatures databases [29]. A total of 884 genes related to PCD pathways including alkaliptosis, apoptosis, autophagy, cuproptosis, disulfidptosis, entotic cell death, ferroptosis, lysosome-dependent cell death, necroptosis, NETotic cell death, oxeiptosis, parthanatos, and pyroptosis were collected for analysis [4].

(Supplementary Table S2). The AUCell algorithm [30] was initially utilized to compute quantitative activation scores for each PCD pathway at the single-cell resolution (Supplementary Fig. S1C). To rigorously translate these continuous enrichment scores into discrete PCD state labels for pseudo-bulk construction, we implemented a mean-based competitive thresholding strategy. Specifically, the global mean AUCell score for each respective pathway across all cells was defined as the activation threshold. Individual cells were then evaluated against these thresholds: cells surpassing the threshold for one or more pathways were uniquely assigned to the specific PCD modality that exhibited the maximum relative activation score. Conversely, cells failing to meet the threshold for any PCD pathway were designated as a basal ‘No death’ state. Thus, the final PCD-state labeling scheme comprised 14 classes in total: 13 named PCD pathways plus one basal ‘No death’ state. Finally, 4096 highly variable genes (HVGs) were selected using the Seurat v3 [31] flavor to construct the input feature matrix.

For the real-world clinical application, integrated bulk microarray and RNA-seq profiles of aging ovaries (GSE307881, GSE154890, GSE253194, GSE235021, GSE289404) were acquired. Unwanted cross-cohort variations were successfully mitigated utilizing the Surrogate Variable Analysis (SVA) [32] algorithm prior to model inference. The efficacy of this batch correction on sample distribution is demonstrated via PCA and median expression box plots (Supplementary Fig. S2A-D).

### Pseudo-bulk generation engine

To train DeepMCD to simultaneously deconvolve cell-type proportions and PCD states, we developed a sophisticated pseudo-bulk generation engine. Unlike traditional uniform sampling, our generator employs a ‘Dropout-Boost’ extreme sampling strategy coupled with a Dirichlet distribution to simulate the severe heterogeneity and potential absence of rare subpopulations inherently found in real clinical tissues [33].

Formally, for each simulated bulk sample, a random subset of cell types is selected. The targeted cellular proportions $\mathrm{p}={\left[{\mathrm{p}}_1,{\mathrm{p}}_2,\dots, {\mathrm{p}}_{\mathrm{K}}\right]}^{\mathrm{T}}$ are sampled from a Dirichlet distribution with a highly skewed concentration parameter $\mathrm{\alpha}$:


(1) \begin{eqnarray*} \mathrm{p}\sim \mathrm{Dir}\left(\mathrm{\alpha} \right), where\ {\mathrm{\alpha}}_{\mathrm{k}}=0.05\ for\ selected\ types \end{eqnarray*}

Given a randomly assigned total cell capacity$\mathrm{m}$(where $300\le \mathrm{m}\le 2000$), the required number of cells for the $\mathrm{k}$-th cell type is determined by ${\mathrm{n}}_{\mathrm{k}}=\mathrm{round}\left({\mathrm{p}}_{\mathrm{k}}\times \mathrm{m}\right)$. Subsequently, individual cells $\left\{{\mathrm{c}}_{\mathrm{k},1},{\mathrm{c}}_{\mathrm{k},2},\dots, {\mathrm{c}}_{\mathrm{k},{\mathrm{n}}_{\mathrm{k}}}\right\}$ are sampled (with or without replacement) from the annotated scRNA-seq pool. The pseudo-bulk expression vector ${\mathrm{x}}_{\mathrm{bulk}}\in{\mathrm{R}}^{4096}$ is calculated as the mean expression of the sampled cells, augmented with Gaussian noise to mimic technical variance:


(2) \begin{eqnarray*} {\mathrm{x}}_{\mathrm{bulk}}=\frac{1}{\mathrm{m}}\sum_{\mathrm{k}=1}^{\mathrm{K}}\sum_{\mathrm{j}=1}^{{\mathrm{n}}_{\mathrm{k}}}{\mathrm{c}}_{\mathrm{k},\mathrm{j}}+\mathrm{\varepsilon}, \mathrm{\varepsilon} \sim \mathrm{N}(0,{\mathrm{\sigma}}^2\mathrm{I}) \end{eqnarray*}

The exact ground-truth proportion vectors for cell types (${\mathrm{Y}}_{\mathrm{cell}}$) and specific PCD states (${\mathrm{Y}}_{\mathrm{pcd}}$) were strictly recorded based on the actual cellular composition of each generated mixture, providing the dual-label supervision required for the multi-task learning framework. In total, 8000 training samples and 1600 validation samples were generated.

### DeepMCD architecture and joint optimization

The DeepMCD network architecture processes the input vector $\mathrm{x}\in{\mathrm{R}}^{\mathrm{B}\times 4096}$ (where $\mathrm{B}$ is the batch size) through task-specific Encoder Blocks comprising Linear layers, LeakyReLU ($\mathrm{\alpha} =0.2$) activations, and Dropout layers ($\mathrm{p}=0.1$). Specifically, the input is decoupled into two distinct latent matrices representing cell types (${\mathrm{H}}_{\mathrm{CT}}$) and PCD patterns (${\mathrm{H}}_{\mathrm{PCD}}$):


(3) \begin{eqnarray*} {\mathrm{H}}_{\mathrm{CT}}=\mathrm{Reshape}\left({\mathrm{MLP}}_{\mathrm{CT}}\left(\mathrm{x}\right)\right)\in{\mathrm{R}}^{\mathrm{B}\times 10\times 256} \end{eqnarray*}

(4) \begin{eqnarray*} {\mathrm{H}}_{\mathrm{PCD}}=\mathrm{Reshape}\left({\mathrm{MLP}}_{\mathrm{PCD}}\left(\mathrm{x}\right)\right)\in{\mathrm{R}}^{\mathrm{B}\times 10\times 256} \end{eqnarray*}

To facilitate cross-task interaction, two learnable class tokens, ${\mathrm{z}}_{\mathrm{CLS}\_\mathrm{CT}}$ and ${\mathrm{z}}_{\mathrm{CLS}\_\mathrm{PCD}}\in{\mathrm{R}}^{1\times 256}$, are prepended to the feature tokens. The joint input sequence ${\mathrm{Z}}^{(0)}$ is further augmented with learnable positional embeddings ${\mathrm{E}}_{\mathrm{pos}}$:


(5) \begin{eqnarray*} {\mathrm{Z}}^{(0)}=\left[{\mathrm{z}}_{\mathrm{CLS}\_\mathrm{CT}}\parallel{\mathrm{z}}_{\mathrm{CLS}\_\mathrm{PCD}}\parallel{\mathrm{H}}_{\mathrm{CT}}\parallel{\mathrm{H}}_{\mathrm{PCD}}\right]+{\mathrm{E}}_{\mathrm{pos}}\in{\mathrm{R}}^{\mathrm{B}\times 22\times 256} \end{eqnarray*}

The core Transformer Encoder consists of $\mathrm{L}=12$ layers with 8 attention heads. Within each layer, multi-head self-attention enables the [CLS_PCD] token to explicitly aggregate contextual features from the CT tokens. The attention mechanism operates as:


(6) \begin{eqnarray*} \mathrm{Attention}\left(\mathrm{Q},\mathrm{K},\mathrm{V}\right)=\mathrm{Softmax}\left(\frac{\mathrm{Q}{\mathrm{K}}^{\mathrm{T}}}{\sqrt{{\mathrm{d}}_{\mathrm{k}}}}\right)\mathrm{V} \end{eqnarray*}

Following the Transformer blocks, the output embeddings corresponding to the class tokens are extracted and passed to task-specific prediction heads. For both CT and PCD tasks, the prediction head adopts a two-layer MLP structure, namely Linear(256, 128) $\to$GELU $\to$Linear(128, output dimension), followed by Softmax normalization to obtain the predicted compositional fractions.

A critical challenge in multi-task learning is the phenomenon of task domination. To address this, DeepMCD implements a homoscedastic uncertainty-based dynamic weighting strategy. The total loss ${\mathrm{L}}_{\mathrm{total}}$ combines the Mean Squared Error (MSE) of both tasks, parameterized by learnable variance scalars ${\mathrm{\sigma}}_1$ and ${\mathrm{\sigma}}_2$:


(7) \begin{eqnarray*} {\mathrm{L}}_{\mathrm{total}}=\exp \left(-{\mathrm{\sigma}}_1\right)\cdotp{\mathrm{L}}_{\mathrm{cell}}+{\mathrm{\sigma}}_1+\exp \left(-{\mathrm{\sigma}}_2\right)\cdotp{\mathrm{L}}_{\mathrm{pcd}}+{\mathrm{\sigma}}_2 \end{eqnarray*}

where ${\mathrm{L}}_{\mathrm{cell}}=\mathrm{MSE}\left({\hat{\mathrm{Y}}}_{\mathrm{cell}},{\mathrm{Y}}_{\mathrm{cell}}\right)$ and ${\mathrm{L}}_{\mathrm{pcd}}=\mathrm{MSE}\left({\hat{\mathrm{Y}}}_{\mathrm{pcd}},{\mathrm{Y}}_{\mathrm{pcd}}\right)$. This strategy is particularly suitable because CT and PCD prediction differ in their target characteristics and optimization behavior, such that a fixed-weight formulation may cause one task to dominate gradient updates. The uncertainty parameters were initialized as a two-element zero tensor and jointly optimized with the backbone network using AdamW (learning rate $=2\times{10}^{-4}$, weight decay $=1\times{10}^{-4}$). Training was performed with a batch size of 128 for up to 150 epochs, with early stopping applied using a patience of 10 epochs.

### Ablation study design via attention masking

To computationally substantiate the biological premise that cellular contextual information is indispensable for high-fidelity PCD state prediction, we implemented a targeted attention-masking ablation strategy. During the forward pass of the Transformer Encoder within the ablated model, a customized attention mask matrix was applied to artificially set the attention weights between the [CLS_PCD] token and all CT tokens to $-\infty$. Post-softmax, these weights effectively become zero, systematically blinding the PCD prediction head to any morphological or cell-type-specific latent representations. The performance degradation, measured by Root Mean Square Error (RMSE) and Lin’s Concordance Correlation Coefficient (CCC), between the full DeepMCD model and the masked ablated model was quantified to assess the magnitude of cross-task guidance.

### Statistical analysis and visualization

All statistical analyses and data visualizations were performed using Python (version 3.12) and R (version 4.2.1) programming environments. For the evaluation of DeepMCD deconvolution performance, Root Mean Square Error (RMSE) and Lin’s Concordance Correlation Coefficient (CCC) were employed as the primary quantitative metrics. In the real-world mouse ovarian aging cohorts, the statistical significance of the dynamic changes in predicted cell-type and PCD proportions across the three chronological age stages was evaluated using the non-parametric Kruskal-Wallis test.

To explore the macroscopic co-occurrence between cellular components and physiological states, Pearson correlation coefficients were calculated, and the resulting network was visualized as a bipartite chord diagram. Furthermore, to investigate clinical relevance, Spearman correlation coefficients were utilized to assess the relationships between the DeepMCD-derived PCD fractions and the expression levels of core ovarian fibrosis-related genes. Both the bipartite chord diagram and the dual-encoded heatmap—concurrently displaying both correlation coefficients (r) and statistical significance (−log10(p)), was generated using customized Python scripts. Throughout this study, a P-value of <0.05 was considered statistically significant.

## Results

### Benchmarking DeepMCD for joint deconvolution on pseudo-bulk datasets

**Figure 2:**
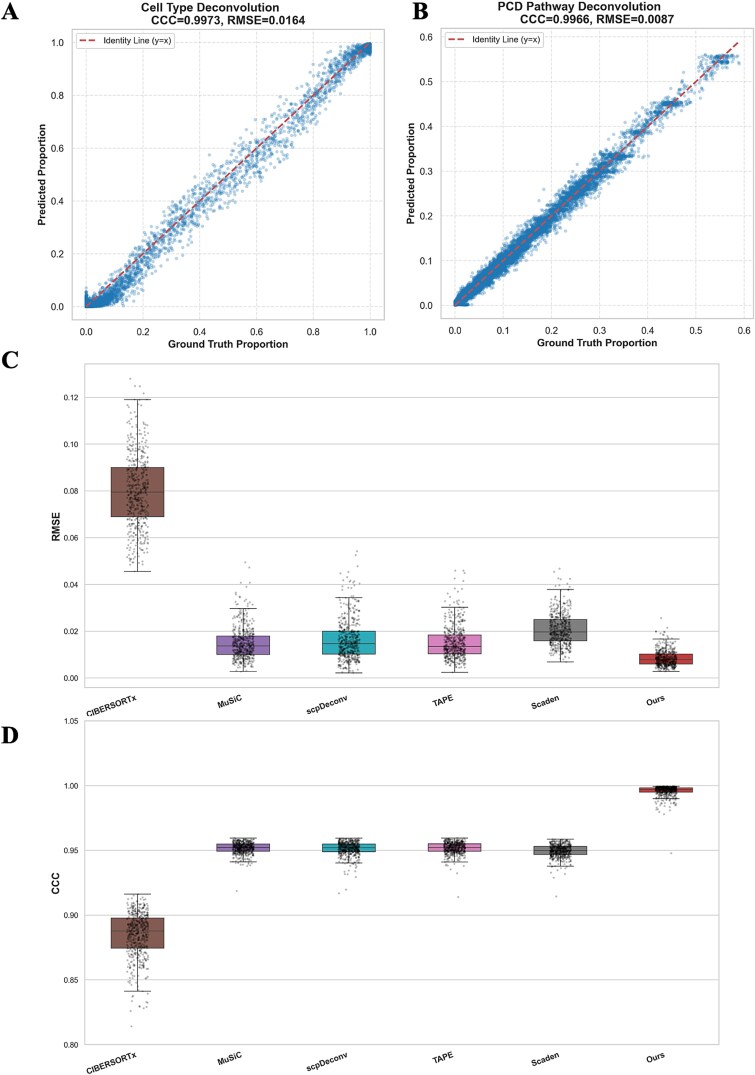
Performance benchmarking of DeepMCD on pseudo-bulk datasets. Scatter plots comparing ground truth versus predicted proportions for (A) 10 cell types and (B) 14 PCD-state classes, illustrating high concordance and low prediction error. (C) RMSE and (D) CCC comparisons evaluate DeepMCD’s overall deconvolution performance against state-of-the-art single-task algorithms.

To systematically evaluate the performance of DeepMCD in simultaneously deconvolving physical cell proportions and dynamic PCD states, we benchmarked the model on a large-scale scRNA-seq-derived pseudo-bulk dataset. DeepMCD demonstrated exceptional accuracy across both predictive tasks. For cell-type deconvolution, the global predicted proportions exhibited a near-perfect concordance with the ground truth, achieving a CCC of 0.9973 and a remarkably low Root Mean Square Error (RMSE) of 0.0164 (Fig. 2A).

Similarly, the model accurately quantified the compositional fractions of the 14 PCD-state classes, consisting of 13 named PCD pathways plus the basal ‘No death’ state, yielding a global CCC of 0.9966 and an RMSE of 0.0087 (Fig. 2B).

For the cell-type deconvolution task, we compared DeepMCD with established reference methods including CIBERSORTx, MuSiC, scpDeconv, TAPE, and Scaden. For the PCD-related task, these methods were used as reference comparators to contextualize performance under the same evaluation framework. DeepMCD significantly outperformed all baseline methods, demonstrating the highest overall CCC and the lowest overall RMSE variance (Fig. 2C, 2D).

Furthermore, we extended this comparative evaluation to the inherently more challenging PCD pathway deconvolution task. As summarized in Table 1, a detailed performance breakdown across the 14 PCD-state classes confirmed that DeepMCD consistently maintained highly robust predictive accuracy. DeepMCD achieved the highest correlation scores across almost all modalities, particularly excelling in challenging pathways such as Parthanatos (0.99), Pyroptosis (0.94), and Disulfidptosis (0.99), systematically outperforming both traditional statistical methods (e.g., CIBERSORT, MuSiC) and deep learning baselines (e.g., Scaden, TAPE). These results strongly suggest that the shared latent representations in DeepMCD successfully prevent negative transfer, allowing the dual tasks to mutually benefit from the joint optimization process.

**Table 1:** Performance comparison (CCC) of PCD-state class deconvolution across different algorithms.

PCD-state class	CIBERSORTx	MuSiC	scpDeconv	TAPE	Scaden	Ours
Alkaliptosis	0.69	0.85	0.85	0.85	0.86	0.99
Apoptosis	0.29	0.57	0.59	0.57	0.74	0.84
Autophagy	0.2	0.85	0.85	0.85	0.85	0.98
Cuproptosis	0.39	0.83	0.83	0.84	0.9	0.96
Disulfidptosis	0.78	0.85	0.85	0.86	0.85	0.99
Entotic cell death	0.54	0.85	0.85	0.85	0.95	0.99
Ferroptosis	0.49	0.8	0.8	0.81	0.85	0.87
Lysosome-dependent cell death	0.69	0.75	0.75	0.75	0.79	0.76
NETotic cell death	0.09	0.82	0.82	0.82	0.46	0.92
Necroptosis	0.49	0.6	0.6	0.59	0.89	0.92
No death	0.69	0.84	0.84	0.84	0.86	0.95
Oxeiptosis	0.59	0.83	0.82	0.82	0.86	0.94
Parthanatos	0.67	0.85	0.85	0.85	0.95	0.99
Pyroptosis	0.09	0.81	0.82	0.81	0.89	0.94

### Comprehensive ablation highlights the necessity of cross-task attention and dynamic weighting

**Figure 3:**
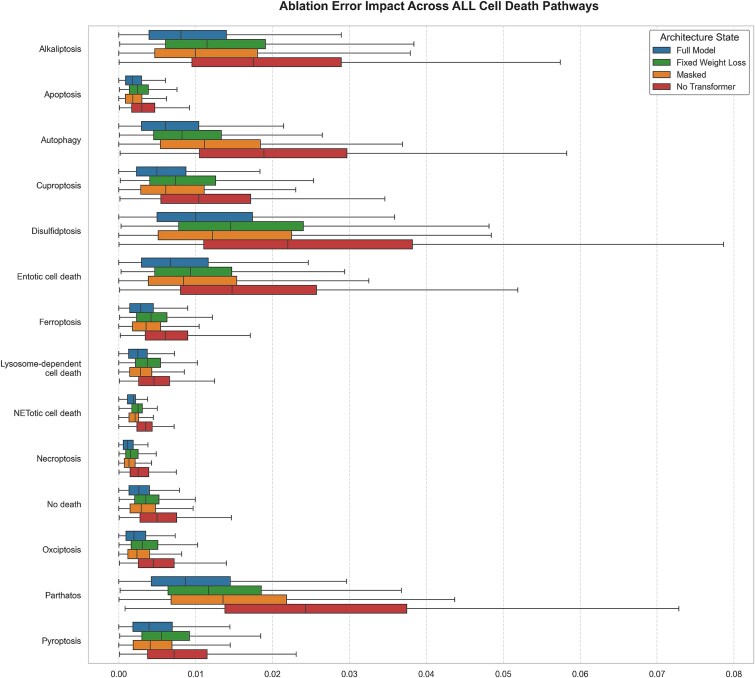
Ablation study highlighting the necessity of cross-task attention and dynamic weighting. Box plots represent the absolute prediction error across the 14 PCD-state classes under four architectural configurations: Full model, fixed weight loss, masked (cell type attention weights constrained to zero), and No transformer (simple vector concatenation).

A core biological premise of DeepMCD is that physiological cell death states are heavily dependent on their specific cell-type contexts. To computationally validate this hypothesis and justify our architectural choices, we performed a rigorous multi-dimensional ablation study, evaluating the absolute prediction error across the 14 PCD-state classes under four distinct model states: Full Model, Fixed Weight Loss, Masked, and No Transformer (Fig. 3).

As illustrated in the ablation impact analysis, the Full Model consistently achieved the lowest error margins. Notably, replacing the dynamic uncertainty-weighting loss with a Fixed Weight Loss resulted in a noticeable increase in prediction errors across almost all pathways, underscoring the necessity of adaptively balancing the dual-task gradients to prevent task domination.

More importantly, the ablation of contextual mechanisms yielded dramatic performance collapses. When we forcibly constrained the attention weights, thereby blinding the PCD prediction head to cellular identity information, the error rates surged significantly. Finally, replacing the Transformer cross-talk module with simple vector concatenation produced the worst performance universally, with massive error spikes observed in Parthanatos, Disulfidptosis, and Autophagy. This pronounced degradation provides compelling algorithmic evidence that simple feature aggregation is insufficient; the multi-head self-attention mechanism is indispensable for dynamically leveraging morphological context to accurately calibrate PCD state predictions.

### Interpretability analysis unveils biologically meaningful latent interactions

Deep learning models are frequently criticized for functioning as opaque ‘black boxes.’ To elucidate the inner workings of DeepMCD and confirm that it learns genuine biological semantics, we visualized both the explicit attention mechanisms and the high-dimensional latent space.

**Figure 4:**
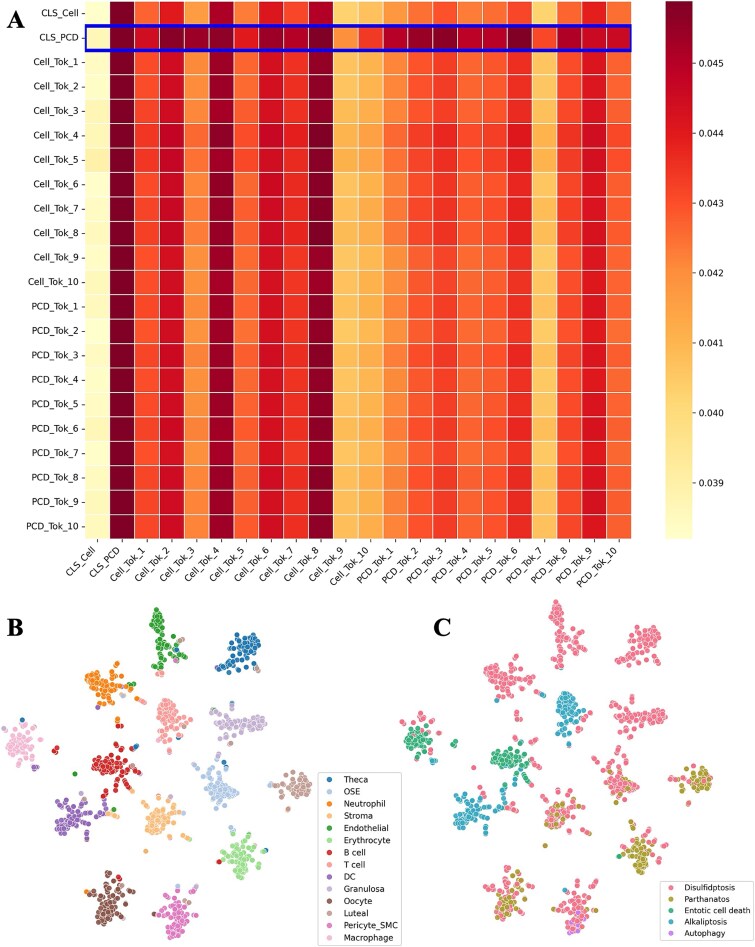
Interpretability of DeepMCD latent interactions. (A) Cross-task attention heatmap extracted from the final transformer layer, demonstrating the explicit querying of cell-type tokens by the [CLS_PCD] token. T-SNE visualization of the [CLS_PCD] latent space aligned by (B) predominant cell lineages and (C) dominant cell death pathways, revealing biologically structured topological clusters.

First, we extracted the attention weight matrix from the final layer of the Transformer encoder. The cross-task attention heatmap visibly demonstrates that the [CLS_PCD] token—responsible for aggregating death state information—assigns robust and non-uniform attention weights across the specific cell-type tokens (Cell_Tok_1 to Cell_Tok_10) (Fig. 4A). This provides direct, transparent evidence that the model explicitly queries distinct cellular morphological features when making decisions regarding PCD fractions.

Furthermore, we visualized the latent space of the [CLS_PCD] embeddings utilizing t-SNE. When aligned by both predominant cell types and cell death pathways, the latent projection revealed a highly structured and biologically coherent topology (Fig. 4B, C). The embeddings naturally clustered into distinct, pure territories that simultaneously mapped to specific cell lineages (e.g., Macrophages, T cells) and their corresponding highly active PCD modalities. This elegant latent alignment supports that DeepMCD successfully untangles the complex transcriptomic mixtures, projecting the raw bulk expression data into a biologically meaningful manifold where cellular identities and physiological death states are fundamentally intertwined.

### DeepMCD recovers the cell-type-specific PCD landscape and prognostic hallmarks in mouse ovarian aging

We applied DeepMCD to real-world bulk transcriptomic cohorts of mouse ovarian aging to decipher the complex interplay between cellular senescence and PCD dynamics. We systematically evaluated the continuous temporal trajectories of both physical cell components and physiological death states across three chronological stages: Young (1-6 M), Middle (6-12 M), and Old (12-25 M).

**Figure 5:**
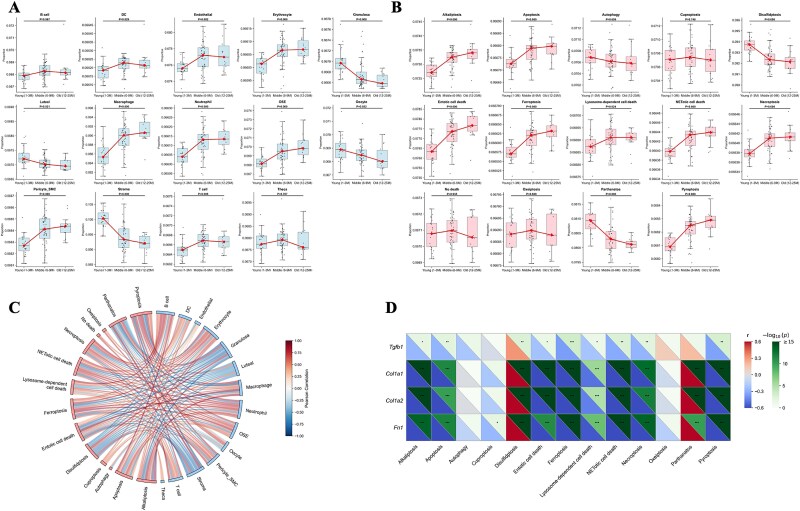
DeepMCD recovers the cell-type-specific PCD landscape and prognostic hallmarks in mouse ovarian aging. (A, B) continuous temporal trajectories of cellular compositions and physiological death states across three chronological aging stages: Young (1-6 M), middle (6-12 M), and old (12-25 M). (C) Bipartite chord diagram illustrating the macroscopic co-occurrence network between specific cell lineages and PCD modalities. (D) Dual-encoded heatmap displaying correlation r and significance -log10(p), correlating DeepMCD-predicted PCD fractions with ovarian fibrosis-related markers (Tgfb1, Col1a1, Col1a2, Fn1).

DeepMCD successfully captured the progressive deterioration of the ovarian microenvironment. Morphologically, the model identified a significant and continuous age-dependent depletion of Oocytes and Granulosa cells, accompanied by a sharp, significant infiltration of inflammatory immune cells, primarily Macrophages and Neutrophils (Fig. 5A). Correspondingly, in the physiological state dimension, DeepMCD revealed a striking transition in cell death execution. Highly inflammatory and lytic PCD modalities—such as Pyroptosis, Apoptosis, Ferroptosis, and Entotic cell death—exhibited significant progressive accumulation over age, while pathways like Parthanatos and Disulfidptosis steadily declined (Fig. 5B).

To further elucidate how these morphological and physiological dimensions orchestrate ovarian aging, we constructed a macroscopic co-occurrence network based on the Pearson correlations of DeepMCD’s dual outputs (Fig. 5C). The network vividly illuminated the dense, highly specific coupling between particular cell lineages and PCD modalities, computationally recovering the phenomena previously observable only via costly single-cell experiments. For instance, Macrophages and Neutrophils demonstrated remarkably strong positive correlations with inflammatory PCD pathways, robustly supporting the hypothesis of macrophage-driven pyroptosis in aging tissues.

We correlated the DeepMCD-predicted PCD fractions with key transcriptomic markers of ovarian fibrosis (Fig. 5D). Utilizing a dual-encoded heatmap, we uncovered profound, divergent correlation patterns. Notably, the predicted proportions of Parthanatos and Disulfidptosis exhibited exceptionally strong positive correlations with the expression levels of core structural genes, including Tgfb1, Col1a1, Col1a2, and Fn1. Conversely, the inflammatory and lytic death modalities that progressively accumulate during aging—such as Pyroptosis, Apoptosis, and Ferroptosis—displayed robust negative correlations with these specific fibrotic markers.

## Discussion

The accurate deconvolution of bulk transcriptomes remains a fundamental challenge in computational biology, primarily due to the inherent confounding of stable physical cellular components with dynamic physiological states. In the context of ovarian aging, this limitation has historically obscured the holistic understanding of how diverse, non-apoptotic PCD modalities orchestrate microenvironmental deterioration. In this study, we developed DeepMCD, an end-to-end multi-task deep learning framework that simultaneously decodes cell-type proportions and PCD compositional fractions. By mapping bulk expression profiles into a shared tokenized latent space and employing a Transformer-based cross-task attention mechanism, DeepMCD successfully bridges the gap between algorithm design and biological priors.

Traditional computational approaches inherently treat the estimation of physical cell fractions and the quantification of dynamic physiological states as mutually exclusive tasks. However, our rigorous ablation studies and interpretability analyses support that physiological functional states are heavily reliant on morphological contexts. By explicitly forcing the [CLS_PCD] token to query cell-type-specific tokens, DeepMCD dynamically leverages cellular identity as a contextual guide, effectively mitigating negative transfer. Furthermore, the integration of a homoscedastic uncertainty-weighting loss ensures balanced multi-task optimization without the need for manual hyperparameter tuning, providing a robust and scalable architecture for complex transcriptomic decoding.

The application of DeepMCD to real-world mouse ovarian aging cohorts offers macroscopic insights into tissue senescence. While classical paradigms heavily emphasize apoptosis as the primary driver of follicular atresia, our model captured a striking progressive accumulation of highly inflammatory and lytic PCD modalities—specifically pyroptosis, ferroptosis, and necroptosis—during chronological aging. Moreover, our computationally derived co-occurrence network robustly linked these inflammatory death executioners directly to the dramatic infiltration of macrophages and neutrophils. This pattern is consistent with the ‘inflammaging’ framework and suggests that the age-dependent increase in inflammatory death modalities, together with myeloid-cell infiltration [34], may contribute to chronic ovarian inflammation and structural deterioration.

Our framework establishes a vital translational link between cell-type-guided PCD fractions and ovarian fibrosis. The dual-encoded correlation analysis revealed that while specific cell death forms are positively associated with core ovarian fibrosis-related genes, the age-accumulated lytic modalities exhibit profound negative correlations. This suggests that the hyperactivation of specific inflammatory PCDs is deeply intertwined with the pathological breakdown or aberrant remodeling of the ovarian fibrotic matrix [35]. Consequently, the specific PCD signatures computationally extracted by DeepMCD from highly accessible bulk RNA-seq data exhibit immense potential as cost-effective, non-invasive candidate digital biomarkers for clinically evaluating ovarian fibrosis and reproductive senescence.

Despite these significant advancements, our study has certain limitations that warrant future investigation. First, the current AUCell-based dominant-label strategy may not fully capture overlapping or transitional cell death programs within individual cells. Therefore, the predicted PCD fractions should be interpreted as dominant-state compositional estimates rather than exhaustive measures of all co-activated death pathways. Second, while DeepMCD captures the compositional coexistence of cell types and PCD states, it cannot resolve their exact physical proximity; integration with emerging spatial transcriptomics [36–40] may help address this limitation. Finally, the current evaluation still relies primarily on pseudo-bulk samples derived from scRNA-seq references and is predominantly limited to mouse datasets. Future work should focus on broader external validation, extension to wider tissue contexts, and refinement of PCD labeling strategies to better capture complex cell-death states.

In conclusion, DeepMCD represents a useful methodological advance in multi-task bulk transcriptome deconvolution. By deeply embedding biological context into deep learning architectures, our study not only provides a highly interpretable computational tool but also unravels the complex, cell-type-specific cell death landscape driving ovarian aging, paving the way for the discovery of novel digital biomarkers and therapeutic targets for age-related pathologies.

**Key points**

- We developed DeepMCD, an end-to-end multi-task deep learning framework that simultaneously deconvolves cell-type proportions and programmed cell death (PCD) fractions from bulk RNA-seq data.
- DeepMCD uses a Transformer-based cross-task attention mechanism to model the dependence of cell death programs on specific cellular contexts, significantly outperforming existing single-task methods.
- Application of DeepMCD to mouse ovarian aging cohorts reconstructed a cell-type-specific PCD landscape and identified a strong association between macrophage enrichment and pyroptosis during ovarian aging.
- DeepMCD-derived PCD fractions showed associations with fibrosis-related genes, supporting their potential as candidate computational biomarkers for ovarian fibrosis and reproductive senescence in mouse ovarian aging.

## Supplementary Material

Supplementary_material_bbag399

## Availability of data and codes

All datasets and source codes in this study are available at https://github.com/ultraypy/DeepMCD

## References

[1] Broekmans FJ, Soules MR, Fauser BC. Ovarian aging: mechanisms and clinical consequences. *Endocr Rev* 2009;30:465–93. 10.1210/er.2009-0006 19589949

[2] Wang S, Zheng Y, Li J et al. Single-cell transcriptomic atlas of primate ovarian aging. *Cell* 2020;180:585–600.e19. 10.1016/j.cell.2020.01.009 32004457

[3] El Khoudary SR, Greendale G, Crawford SL et al. The menopause transition and Women’s health at midlife: a progress report from the study of Women’s health across the nation (SWAN). *Menopause* 2019;26:1213–27. 10.1097/GME.0000000000001424 31568098 PMC6784846

[4] Galluzzi L, Vitale I, Aaronson SA et al. Molecular mechanisms of cell death: recommendations of the nomenclature committee on cell death 2018. *Cell Death Differ* 2018;25:486–541. 10.1038/s41418-017-0012-4 29362479 PMC5864239

[5] Tilly JL . Commuting the death sentence: how oocytes strive to survive. *Nat Rev Mol Cell Biol* 2001;2:838–48. 10.1038/35099086 11715050

[6] Shi J, Zhao Y, Wang K et al. Cleavage of GSDMD by inflammatory caspases determines pyroptotic cell death. *Nature* 2015;526:660–5. 10.1038/nature15514 26375003

[7] Dixon SJ, Lemberg KM, Lamprecht MR et al. Ferroptosis: an iron-dependent form of nonapoptotic cell death. *Cell* 2012;149:1060–72. 10.1016/j.cell.2012.03.042 22632970 PMC3367386

[8] Franceschi C, Garagnani P, Parini P et al. Inflammaging: a new immune-metabolic viewpoint for age-related diseases. *Nat Rev Endocrinol* 2018;14:576–90. 10.1038/s41574-018-0059-4 30046148

[9] Liu X, Zhao Y, Feng Y et al. Ovarian aging: mechanisms, age-related disorders, and therapeutic interventions. *MedComm (2020)* 2025;6:e70481. 10.1002/mco2.70481 41256733 PMC12620568

[10] Stuart T, Satija R. Integrative single-cell analysis. *Nat Rev Genet* 2019;20:257–72. 10.1038/s41576-019-0093-7 30696980

[11] Denisenko E, Guo BB, Jones M et al. Systematic assessment of tissue dissociation and storage biases in single-cell and single-nucleus RNA-Seq workflows. *Genome Biol* 2020;21:130. 10.1186/s13059-020-02048-6 32487174 PMC7265231

[12] Lafzi A, Moutinho C, Picelli S et al. Tutorial: guidelines for the experimental Design of Single-Cell RNA sequencing studies. *Nat Protoc* 2018;13:2742–57. 10.1038/s41596-018-0073-y 30446749

[13] Stark R, Grzelak M, Hadfield J. RNA sequencing: the teenage years. *Nat Rev Genet* 2019;20:631–56. 10.1038/s41576-019-0150-2 31341269

[14] Newman AM, Steen CB, Liu CL et al. Determining cell type abundance and expression from bulk tissues with digital cytometry. *Nat Biotechnol* 2019;37:773–82. 10.1038/s41587-019-0114-2 31061481 PMC6610714

[15] Avila Cobos F, Vandesompele J, Mestdagh P et al. Computational deconvolution of transcriptomics data from mixed cell populations. *Bioinformatics* 2018;34:1969–79. 10.1093/bioinformatics/bty019 29351586

[16] Menden K, Marouf M, Oller S et al. Deep learning-based cell composition analysis from tissue expression profiles. *Sci Adv* 2020;6:eaba2619. 10.1126/sciadv.aba2619 32832661 PMC7439569

[17] Racle J, de Jonge K, Baumgaertner P et al. Simultaneous enumeration of cancer and immune cell types from bulk tumor gene expression data. *Elife* 2017;6:e26476. 10.7554/eLife.26476 29130882 PMC5718706

[18] Zhao T, Liu R, Sun Y et al. DECODE: deep learning-based common deconvolution framework for various omics data. *Nat Methods* 2026;23:596–608. 10.1038/s41592-026-03007-y 41772096 PMC12982128

[19] Barbie DA, Tamayo P, Boehm JS et al. Systematic RNA interference reveals that oncogenic KRAS-driven cancers require TBK1. *Nature* 2009;462:108–12. 10.1038/nature08460 19847166 PMC2783335

[20] Hänzelmann S, Castelo R, Guinney J. GSVA: gene set variation analysis for microarray and RNA-Seq data. *BMC Bioinformatics* 2013;14:7. 10.1186/1471-2105-14-7 23323831 PMC3618321

[21] Frank D, Vince JE. Pyroptosis versus necroptosis: similarities, differences, and crosstalk. *Cell Death Differ* 2019;26:99–114. 10.1038/s41418-018-0212-6 30341423 PMC6294779

[22] Vaswani A, Shazeer N, Parmar N et al. Attention is all you need. In: Proceedings of the Advances in Neural Information Processing Systems, Vol. 30. Curran Associates, Inc, 2017.

[23] Cipolla R, Gal Y, Kendall A. Multi-task learning using uncertainty to weigh losses for scene geometry and semantics. In: Proceedings of the 2018 IEEE/CVF Conference on Computer Vision and Pattern Recognition, pp. 7482–91, 2018.

[24] Edgar R, Domrachev M, Lash AE. Gene expression omnibus: NCBI gene expression and hybridization Array data repository. *Nucleic Acids Res* 2002;30:207–10. 10.1093/nar/30.1.207 11752295 PMC99122

[25] Korsunsky I, Millard N, Fan J et al. Fast, sensitive and accurate integration of single-cell data with harmony. *Nat Methods* 2019;16:1289–96. 10.1038/s41592-019-0619-0 31740819 PMC6884693

[26] Kanehisa M, Goto S. KEGG: Kyoto Encyclopedia of genes and genomes. *Nucleic Acids Res* 2000;28:27–30. 10.1093/nar/28.1.27 10592173 PMC102409

[27] Stelzer G, Rosen N, Plaschkes I et al. The GeneCards suite: from gene data mining to disease genome sequence analyses. *Curr Protoc Bioinformatics* 2016;54:1.30.1–33. 10.1002/cpbi.5 27322403

[28] Fabregat A, Jupe S, Matthews L et al. The Reactome pathway knowledgebase. *Nucleic Acids Res* 2018;46:D649–55. 10.1093/nar/gkx1132 29145629 PMC5753187

[29] Liberzon A, Birger C, Thorvaldsdóttir H et al. The molecular signatures database (MSigDB) Hallmark gene set collection. *Cell Syst* 2015;1:417–25. 10.1016/j.cels.2015.12.004 26771021 PMC4707969

[30] Aibar S, González-Blas CB, Moerman T et al. SCENIC: single-cell regulatory network inference and clustering. *Nat Methods* 2017;14:1083–6. 10.1038/nmeth.4463 28991892 PMC5937676

[31] Stuart T, Butler A, Hoffman P et al. Comprehensive integration of single-cell data. *Cell* 2019;177:1888–1902.e21. 10.1016/j.cell.2019.05.031 31178118 PMC6687398

[32] Leek JT, Johnson WE, Parker HS et al. The Sva package for removing batch effects and other unwanted variation in high-throughput experiments. *Bioinformatics* 2012;28:882–3. 10.1093/bioinformatics/bts034 22257669 PMC3307112

[33] Jew B, Alvarez M, Rahmani E et al. Accurate estimation of cell composition in bulk expression through robust integration of single-cell information. *Nat Commun* 2020;11:1971. 10.1038/s41467-020-15816-6 32332754 PMC7181686

[34] Sayed N, Huang Y, Nguyen K et al. An inflammatory aging clock (iAge) based on deep learning tracks multimorbidity, Immunosenescence. *Frailty and Cardiovascular Aging Nat Aging* 2021;1:598–615. 10.1038/s43587-021-00082-y 34888528 PMC8654267

[35] Amargant F, Manuel SL, Tu Q et al. Ovarian stiffness increases with age in the mammalian ovary and depends on collagen and hyaluronan matrices. *Aging Cell* 2020;19:e13259. 10.1111/acel.13259 33079460 PMC7681059

[36] Marx V . Method of the year: spatially resolved transcriptomics. *Nat Methods* 2021;18:9–14. 10.1038/s41592-020-01033-y 33408395

[37] Jia Y, Liu J, Chen L et al. THItoGene: a deep learning method for predicting spatial transcriptomics from histological images. *Brief Bioinform* 2024;25:bbad464. 10.1093/bib/bbad464 PMC10749789 38145948

[38] Mu J, Yao Y, Chen Q et al. GATCL: graph attention network meets contrastive learning for spatial domain identification. *Brief Bioinform* 2026;27:bbag043. 10.1093/bib/bbag043 41678736 PMC12900075

[39] Liu X, Lü X, Chen Q et al. PanGIA: a universal framework for identifying association between ncRNAs and diseases. *GigaScience* 2025;14:giaf123. 10.1093/gigascience/giaf123 41105014 PMC12532321

[40] Jia Y, Dong H, Li L et al. xQTLatlas: a comprehensive resource for human cellular-resolution multi-omics genetic regulatory landscape. *Nucleic Acids Res* 2025;53:D1270–7. 10.1093/nar/gkae837 39351883 PMC11701524

