# Supplementary material for: Cell type–specific dissection of cell death programs during ovarian aging
Source: Brief Bioinform. 2026 Jul 30;27(4):bbag399. doi: 10.1093/bib/bbag399 (PMC13435229; doi:10.1093/bib/bbag399)
Supplement: Supplementary file 1 [file supplementary_material_bbag399.zip › Supplementary_Data.docx]

# Supplementary Data

Supplementary Figure 1. Single-cell seq data preprocessing and programmed cell death (PCD) pathway analysis. (A, B) UMAP projection and dot plot of cell-type-specific marker genes. (C) Violin plots showing the distribution of AUCell scores for 13 named PCD pathways (from left to right: Alkaliptosis, Apoptosis, Autophagy, Cuproptosis, Disulfidptosis, Entotic cell death, Ferroptosis, Lysosome-dependent cell death, NETotic cell death, Necroptosis, Oxeiptosis, Parthanatos, Pyroptosis).

**Alternative text:**
Supplementary Figure 1 shows single-cell RNA-seq preprocessing results, including UMAP visualization of annotated ovarian cell types, marker-gene expression patterns, and AUCell score distributions for 13 named PCD pathways.

Supplementary Figure 2. Bulk-seq data preprocessing (A, B) PCA plot before/after batch correction(C, D)Box plot of sample median expression by dataset before/after correction.

**Alternative text:**
Supplementary Figure 2 shows bulk transcriptomic preprocessing results, including PCA plots and sample median-expression box plots before and after batch correction.

Supplementary Table 1. The essential information of the bulk-RNA datasets and scRNA-seq datasets included in this study.

| GEO | Samples | | | |
| --- | --- | --- | --- | --- |
|  | Total | Young | Middle | Aging |
| GSE307881 | 18 | 13 | 5 | 0 |
| GSE154890 | 20 | 10 | 5 | 5 |
| GSE253194 | 18 | 6 | 3 | 9 |
| GSE235021 | 24 | 9 | 15 | 0 |
| GSE289404 | 6 | 6 | 0 | 0 |
| GSE200612 (scRNA-seq) | 9 | 5 | 2 | 2 |
| GSE232309 (scRNA-seq) | 8 | 4 | 4 | 0 |
| GSE236712 (scRNA-seq) | 2 | 1 | 0 | 1 |
| GSE317144 (scRNA-seq) | 4 | 2 | 0 | 2 |
